# Pediatric video capsule endoscopy: diagnostic yield, safety, and clinical impact in a tertiary center

**DOI:** 10.3389/fped.2026.1817488

**Published:** 2026-04-14

**Authors:** Maya Granot, Nurit Nachum, Alexander Krauthammer, Tal David Berger, Shomron Ben-Horin, Uri Kopylov, Batia Weiss, Yael Haberman

**Affiliations:** 1 Division of Pediatric Gastroenterology and Nutrition, Edmond and Lily Safra Children’s Hospital, Sheba Medical Center, Tel-Hashomer, Israel; 2 The Gray Faculty of Medical and Health Sciences, Tel-Aviv University, Tel-Aviv, Israel; 3 Department of Gastroenterology, Sheba Medical Center, Tel-Hashomer, Israel; 4 Department of Pediatrics, Cincinnati Children’s Hospital Medical Center and the University of Cincinnati College of Medicine, Cincinnati, OH, United States

**Keywords:** capsule endoscopy, children, imaging, inflammatory bowel disease, small bowel

## Abstract

**Introduction:**

Video capsule endoscopy (VCE) enables direct, radiation-free visualization of the small bowel mucosa and is endorsed by pediatric guidelines as a key tool in the evaluation of Crohn's disease (CD). Despite this, VCE remains underused in routine pediatric practice, and its real-world clinical impact is insufficiently characterized. We aimed to assess the diagnostic yield, safety, and management consequences of pediatric VCE in a tertiary center and to compare its findings with cross-sectional imaging and biomarkers.

**Methods:**

We conducted a retrospective, single-center study of VCE procedures in children younger than 18 years performed between 2018 and 2024. Demographic, clinical, imaging, and laboratory data were reviewed to characterize indications, safety, and clinical yield.

**Results:**

Seventy-six VCE examinations were performed in 60 children (mean age 14.8 years; 41% female). Endoscopic placement was required in 19 patients (25%). Dissolvable patency capsule testing to evaluate non-retention of the real VCE was performed in 22/80 (27.5%) planned VCEs, with four failures that abrogated further application of VCE. The main indication for VCE was suspected or established CD (57 VCEs in 45 children); other indications included polyposis syndromes, eosinophilic gastrointestinal disease, iron deficiency anemia, and gastrointestinal bleeding. In the CD subgroup, VCE supported a new diagnosis in 13 of 28 cases (46%) and prompted disease reclassification in 12 of 29 cases (41%). Small bowel inflammation was noted in 35 of 42 VCEs (83%) in new or known patients with CD [Lewis score (LS) > 135], with a median LS of 563. VCE findings led to the initiation or escalation of CD treatment in 22 of 42 patients (52%). One capsule retention occurred, revealing a previously unsuspected severe stricturing (B2) phenotype and leading to a planned, nonurgent intestinal resection. Among the 46 children who underwent both VCE and cross-sectional imaging, concordance between VCE and MRE/IUS was modest (*κ* = 0.07, 95% CI −0.19 to 0.34), underscoring the complementary value of VCE.

**Conclusion:**

VCE is a safe and well-tolerated modality for evaluating pediatric small bowel disease, particularly CD, and frequently reveals clinically relevant inflammation missed by conventional imaging. These findings support its broader integration into pediatric practice.

## Introduction

Video capsule endoscopy (VCE) is a noninvasive technique for direct visualization of the small bowel mucosa. VCE enables high-resolution mucosal imaging without sedation or radiation exposure, overcoming the limitations of conventional endoscopy. Adult patient surveys have indicated that most individuals prefer VCE over colonoscopy or MRE because of its comfort and lower anxiety burden (1). The PillCam SB received FDA approval for adults in 2001, for children aged 10–18 years in 2004, and from age 2 in 2009 (2–4). Studies and case reports have shown safe and feasible use in even younger children, including infants as young as 8 months, 7.9 kg, with no serious complications (5, 6).

In pediatric gastroenterology, VCE is mainly used for suspected or established CD, obscure gastrointestinal bleeding, iron deficiency anemia, and polyposis syndromes (6–8). Early studies showed feasibility with completion rates over 85% and capsule retention rates under 2% (5, 9, 10). Recent multicenter studies demonstrate diagnostic yields of 40%–80%, especially for proximal small bowel lesions, leading to management changes in about one third of cases (11–15). Moreover, longitudinal pediatric studies reveal that mucosal healing observed with VCE correlates with better disease outcomes (11). Compared with Magnetic resonance enterography (MRE) or Intestinal ultrasound (IUS), VCE offers higher sensitivity for mucosal inflammation in proximal disease and serves as a complementary tool when combined with imaging (12, 16). For children unable to swallow, endoscopic deployment under sedation provides a reliable alternative with no significant complications, even in infants (5, 17, 18).

Current guidelines from the European Society of Gastrointestinal Endoscopy (ESGE) (19, 20) and the NASPGHAN/ESPGHAN pediatric consortium (21) endorse VCE as a first-line or adjunctive tool for small bowel evaluation in CD, obscure gastrointestinal bleeding, and polyposis, recommending patency testing in at-risk patients and endoscopic insertion for those unable to swallow (19–22). Despite guideline endorsements and growing pediatric experience, small-bowel capsule endoscopy is still used selectively in children, and real-world data on its diagnostic yield, safety, and impact on management in pediatric Crohn's disease remain limited (21). This gap is particularly relevant given the increasing emphasis on treat-to-target strategies and mucosal healing, highlighting the need for studies evaluating its real-world effectiveness and clinical impact. Therefore, we aimed to evaluate the diagnostic yield, safety, and clinical impact of pediatric VCE in a tertiary center, with a specific focus on children with suspected or established Crohn's disease and on its agreement with cross-sectional imaging and biomarkers.

## Methods

### Ethics statement

The study was approved by the institutional ethics committee of Sheba Medical Center, Tel Hashomer, Israel, which waived the requirement for written informed consent due to the retrospective design.

### Cohort

This retrospective single-center study included all video capsule endoscopy (VCE) procedures performed in children aged 18 years or younger at Sheba Medical Center, a tertiary pediatric gastroenterology unit, between January 2018 and December 2024. Demographic, clinical, laboratory, treatments, endoscopic, and imaging data were extracted from electronic medical records and endoscopy databases using a standardized case report form.​ Data collected included age, sex, and anthropometric measurements at the time of the test. For patients with CD, disease location and phenotype were classified according to the Paris classification before VCE, and disease duration and age at CD diagnosis were recorded. Laboratory data within two months of the VCE included fecal calprotectin (FC), C-reactive protein (CRP), albumin, and complete blood count, when available.​Some children underwent more than one VCE during the study period.

### VCE examinations

All VCE examinations were performed using the PillCam SB system (Medtronic, Minneapolis, MN, USA), according to institutional protocols. Children were able to swallow the ingested capsule with water after an overnight fast, whereas those unable to swallow underwent endoscopic deployment into the gastric antrum or duodenum under sedation. Use of a patency capsule before VCE was at the discretion of the treating physician, based on clinical and radiologic suspicion of stricturing disease or prior surgery. Completion of the study was defined as visualization of the cecum or ileocecal valve within the recording time, and gastric and small bowel transit times were calculated from the recorded images. Images were reviewed on dedicated software by experienced gastroenterologists with expertise in VCE. VCE studies were classified as “abnormal” if they demonstrated mucosal abnormalities like polyps, ulcerations, erosions, edema, erythema, strictures, or active bleeding consistent with clinically relevant pathology. Small bowel inflammatory activity was quantified using the Lewis score (LS), calculated for the most affected tertile, and categorized as no significant inflammation (LS 0–135), mild (LS 135–790), or moderate–severe (LS ≥790). A Lewis score greater than 135 indicates at least mild small-bowel inflammatory activity was considered abnormal, while examinations with a Lewis score of 0–135 were classified as normal.

For patients with CD, notes were reviewed to capture predefined clinical outcomes after VCE: new CD diagnosis, change in disease classification (including L4 status), treatment initiation or escalation, de-escalation, additional investigations, or surgical referral. Standard EGD/ileocolonoscopy was performed in all patients evaluated for suspected CD as part of routine diagnostic work-up, but those procedures were not always performed at the time of VCEs. Children who required endoscopic capsule placement, therefore, underwent a separate procedure under anesthesia specifically for capsule insertion. Treatment changes were identified by reviewing the treating physicians' clinic notes and medication records. We considered a change to be VCE-driven when the physician explicitly documented that the VCE findings prompted treatment initiation, escalation, de-escalation, switch in therapy, or surgical referral, and we only recorded changes that occurred within a predefined time window after the VCE examination.‏

### Imaging and biomarkers

In line with the primary study aim, comparative analyses of cross-sectional imaging were restricted to children investigated for suspected or established CD, in whom MRE and IUS performed within two months of VCE were retrieved when available, whereas imaging obtained for other VCE indications (for example, CT or nuclear medicine studies) was not included in these concordance analyses. MRE and IUS performed within two months of VCE were retrieved when available and categorized as “active” or “no active disease” based on standardized radiologic reports. Biochemical remission was defined as FC ≤ 100 µg/g and CRP within the local laboratory reference range, radiologic remission as absence of active inflammation on MRE or IUS, and endoscopic/VCE remission as LS < 135.

### Statistics

Continuous variables are presented as mean and standard deviation (SD) or median and interquartile range (IQR), according to distribution, and categorical variables as counts and percentages. Group comparisons used Student's *t*-test or Mann–Whitney *U*-test for continuous variables and chi-square or Fisher's exact test for categorical variables, as appropriate. Agreement between VCE and MRE or IUS regarding the presence of active small bowel inflammation was quantified using Cohen's kappa, and McNemar's test was applied to compare paired proportions. A two-sided *p*-value <0.05 was considered statistically significant.

## Results

### Study population

**Figure 1:**
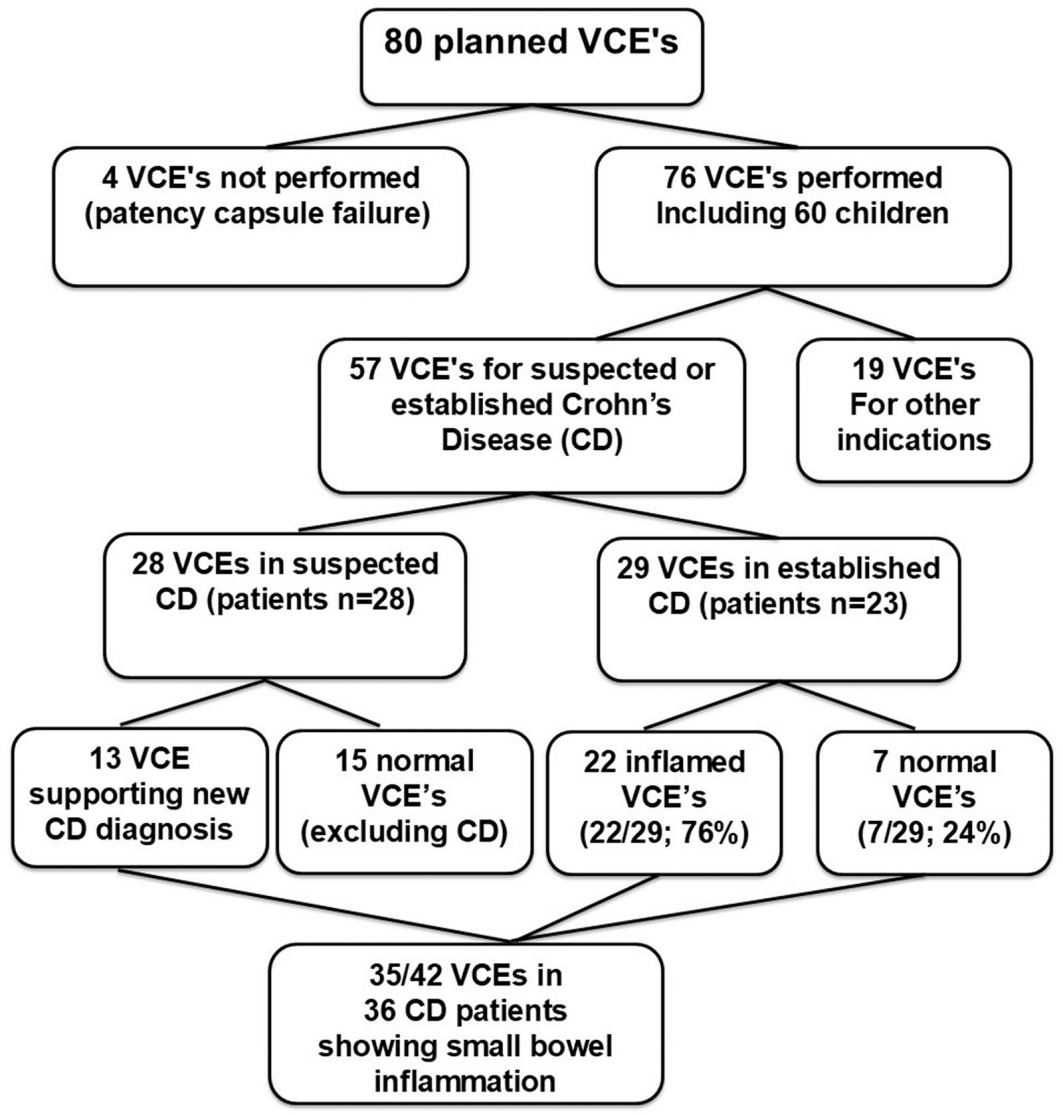
Flow chart of the pediatric video capsule endoscopy cohort. The diagram depicts all 76 VCE examinations performed in 60 children, including 57 examinations in children with suspected or established Crohn's disease and 19 examinations for other indications.

Seventy-six VCE examinations were performed in 60 children (mean age 14.8 years; 41% female) during the study period between 2018 and 2024 (Figure 1 and Table 1). Of the performed 76 VCEs, most (57/76) were performed in children with suspected or established CD, and 19 tests were performed for other indications, including polyposis syndromes, eosinophilic gastrointestinal disease, anemia, and obscure gastrointestinal bleeding.

**Table 1:** Demographic and procedural characteristics.

Variable	All VCEs	VCEs done for suspected and established CD
VCE, *n*	*n* = 76	*n* = 57
Patients, *n*	*n* = 60	*n* = 45
Female	25/60 (42%)	19/45 (42%)
Age at VCE, years Mean (SD)	14.8 (3.5)	15.8 (2.5)
Weight at VCE (kg) Median (IQR)	52 (35, 70)	65 (51–70)
Weight (kg) Range at VCE	15, 110	19.7,110
Height at VCE (cm) Median (IQR)	160 (148, 172)	177 (168–184)
Gastric passage time, minutes Median (IQR)	15 (8, 58)	16 (10, 65)
Small bowel passage time, minutes Median (IQR)	249 (183, 324)	247 (181, 324)
Patency capsule use	22 (28%)	19 (32%)
Endoscopic insertion of VCE	19 (25%)	10 (17%)

VCE, video capsule endoscopy; CD, Crohn's disease; SD, Standard deviation IQR, interquartile range. Data are presented per VCE examination except for gender that is presented per subjects as specified.

### VCE performance and safety

Endoscopic capsule placement under sedation was required in 19 of 76 procedures (25%), primarily in children unable to swallow the capsule. Dissolvable patency capsule testing before using the VCE to evaluate potential retention of VCE was performed in 22/80 (27.5%) planned VCEs, with four failures that abrogated further application of VCE and prevented potential capsule retention. Interestingly, the use of patency capsules increased to 40% of eligible cases in 2023–2024, highlighting their inclusion in clinical practice in recent years. One capsule retention occurred, indicating a non-suspected severe stricture(B2) disease phenotype, and led to the decision for a nonurgent intestinal resection, performed two months later, without long-term sequelae. The median small bowel passage time was 249 min (IQR 183–324), with no relevant difference between CD and non-CD indications (Table 1).

### Diagnostic and therapeutic management yields in CD, including establishing a new diagnosis, re-classifying disease extent, or modifying treatment

Among the 57 CD-related VCEs, 28 were performed in patients suspected of having CD, and 29 in those with established disease (Table 2). Among children investigated for suspected CD, VCE supported a new diagnosis in 13 of 28 tests (46%) (Table 2). Diagnosis was based on typical small-bowel inflammatory lesions and concordant clinical and laboratory findings.

**Table 2:** Laboratory results, VCE characteristics, and cross-sectional imaging findings among children with suspected or established crohn's disease who underwent video capsule endoscopy.

Variable	VCEs in suspected or established CD (*n* = 57)	VCEs in new and established CD (*n* = 42)
Lab results around VCE		
Fecal calprotectin (microg/gr) Median (IQR)	154 (40, 391)	155 (51,450)
CRP (mg/L) Median (IQR)	1 (1, 7)	1 (1,7)
Albumin (g/dL) Mean (SD)	4.21 (0.38)	4.2 (0.4)
Imaging around VCE		
MRE findings	*n* = 42	*n* = 27
Abnormal	15 (36%)	10 (37%)
Normal	27 (64%)	17 (63%)
IUS findings	*n* = 21	*n* = 18
Abnormal	9 (43%)	8 (44%)
Normal	12 (57%)	10 (56%)
VCE LS score Median (IQR)	563 (225, 903)	563 (225,903)
LS, 0–135 *n*, (%)	24 (42%)	7 (17%)
LS, 135–790, *n*, (%)	17 (30%)	19 (45%)
LS, >790, *n*, (%)	16 (28%)	16 (38%)

CD, Crohn's disease; FC, Fecal Calprotectin; IQR, interquartile range; CRP, C-reactive protein; MRE, magnetic resonance enterography; IUS, intestinal ultrasound; VCE,video capsule endoscopy; LS, Lewis score. Data are presented per VCE examination.

**Figure 2:**
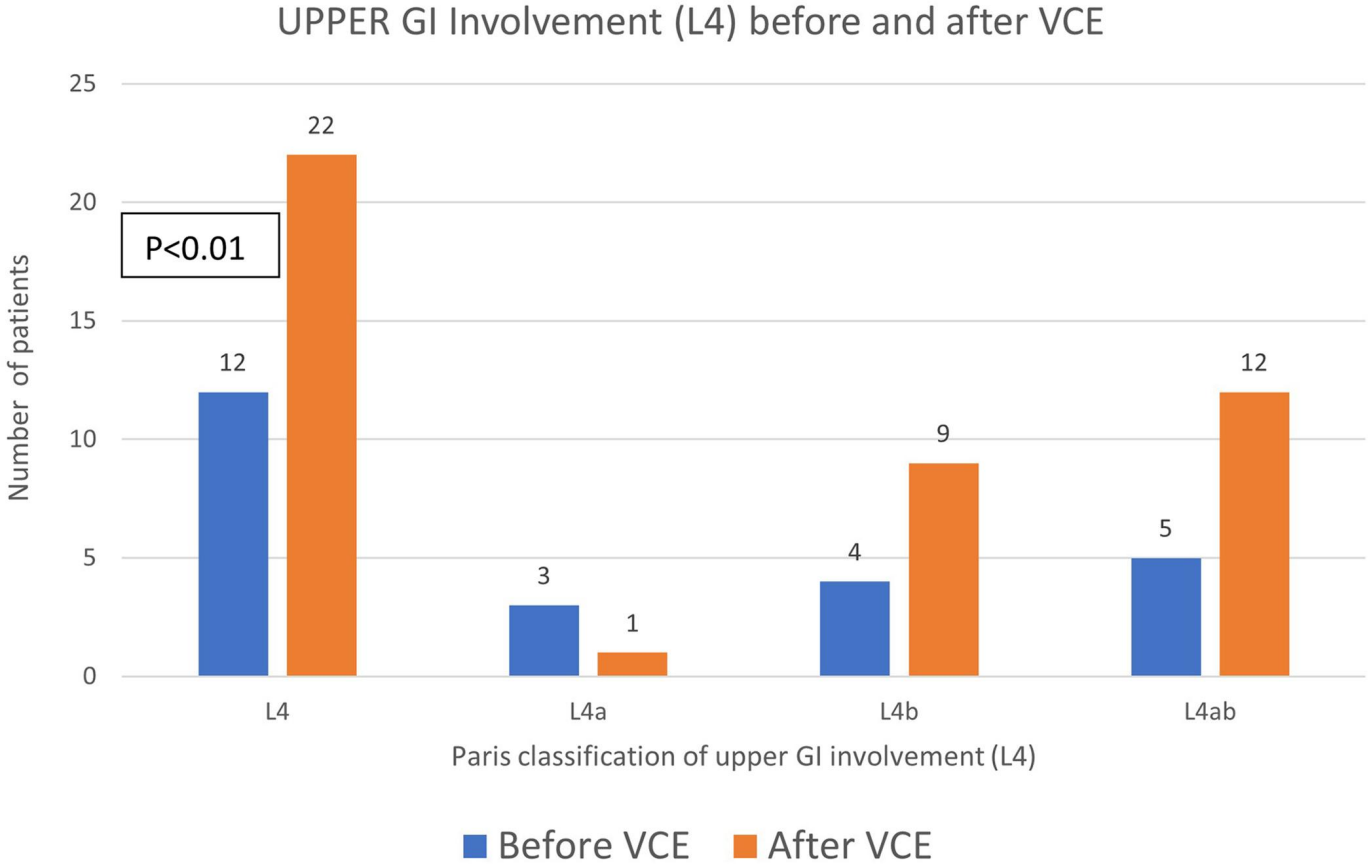
Change in Paris L4 upper gastrointestinal classification before and after video capsule endoscopy. Change in Paris L4 upper gastrointestinal classification before and after video capsule endoscopy in 23 children with established Crohn's disease. Blue bars show the number of children in each L4 subgroup before VCE, and orange bars show the number after VCE.

Characteristics of the group of children with established CD (Table 3, 42 VCEs examinations in 36 patients), including children with newly diagnosed and established CD and excluding children with suspected CD who were ultimately not diagnosed with CD, were as follows: mean age at CD diagnosis of 13.8 years (SD 3.9), median disease duration at VCE of 11.6 months (IQR 0–32), L1 ileal involvement in 83%, and 68% were treated with biologic therapy at the time of the test. Within established and new CD related VCEs (*n* = 42, Table 2), small bowel inflammation was noted in 35 tests (83%), with a median LS of 563 (IQR 225–903); 7 studies (17%) showed no significant small bowel inflammation (LS ≤ 135), 19 (45%) indicated mild activity (LS 135–790), and 16 (38%) showed moderate–severe activity (LS ≥ 790). In the established CD group, before VCE disease was classified as L4 in 30.4% (*n* = 7/23), increasing to 73.9% of patients (*n* = 17/23, Figure 2, *p* = 0.01) after VCE evaluation.

**Table 3:** Clinical characteristics, disease location, phenotype, and treatment among children with established and newly diagnosed Crohn’s disease undergoing video capsule endoscopy.

Variable	Established CD	New CD
VCEs, *n*	29	13
Patients, *n*	23	13
Disease duration, months Mean (SD)	24 (32)	new diagnosis
Age at diagnosis, years Mean (SD)	13.8 (3.9)	15.4 (2.7)
Disease Location at diagnosis		
Ileum (L1) *n* (%)	19 (83%)	10 (77%)
Colonic (L2) *n* (%)	2 (8.5%)	0 (0%)
Ileo-colonic (L3) *n* (%)	2 (8.5%)	0 (0%)
Upper GI involvement (L4) *n* (%)	7 (30.4%)	10 (77%)
L4a	2 (9%)	1 (8%)
L4b	2 (9%)	3 (23%)
L4ab	3 (13%)	6 (46%)
Perianal involvement *n* (%)	2 (9%)	0 (0%)
Disease phenotype		
B1 inflammatory *n* (%)	22 (96%)	13 (100%)
B2 stricturing *n* (%)	1 (4%)	0 (0%)
B3 penetrating *n* (%)	0 (0%)	0 (0%)
Treatment during VCE	*n* = 22	new diagnosis
Nutrition *n* (%)	3 (14%)	–
5-ASA *n* (%)	1 (4.5%)	–
Azathioprine *n* (%)	2 (9%)	–
Methotrexate	1 (4.5%)	–
Biologics	15 (68%)	–
Infliximab *n* (%)	4 (28%)	
Adalimumab *n* (%)	8 (36%)	
Ustenkinumab *n* (%)	1 (4.5%)	
Risankizumab *n* (%)	2 (9%)	

CD, Crohn's disease; VCE, video capsule endoscopy; SD, standard deviation; IQR, interquartile range; 5-ASA, 5-aminosalicylic acid. Data are presented per patient unless otherwise specified.

Within the 13 new and 23 established patients with CD, VCE-driven decisions included treatment initiation or escalation in 21 of 36 patients (58%). Among patients with established CD, treatment was initiated or intensified in 12/23 patients (52%). Among newly diagnosed patients with CD, VCE prompted treatment initiation in 9 of 13 patients (69%), including 5 who received pharmacologic therapy and 4 who started partial enteral nutrition. In addition, VCE results informed surgical referral in the single retention case and helped avoid unnecessary procedures in patients without detectable small bowel pathology, directly affecting the management of 23/36 (64%) patients with CD.

### Correlation with imaging and biomarkers

**Figure 3:**
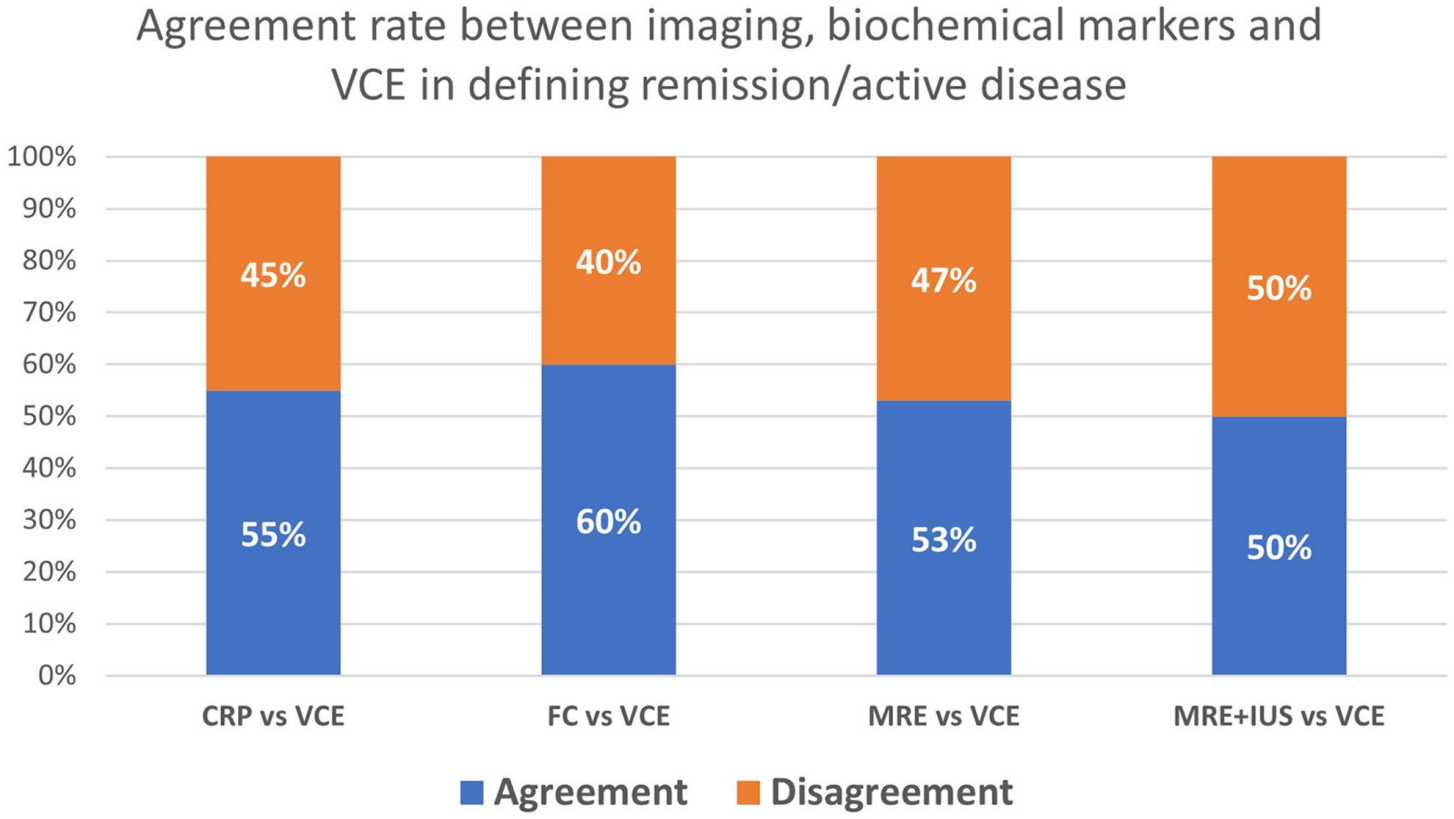
Agreement between biochemical markers, cross-sectional imaging, and video capsule endoscopy in classifying remission and active disease among 57 VCE examinations performed in children with suspected or established Crohn's disease. Blue bars indicate agreement with VCE and orange bars indicate disagreement for CRP, fecal calprotectin, magnetic resonance enterography, and combined magnetic resonance enterography plus intestinal ultrasound.

In the overall subgroup of suspected and established CD with both VCE and MRE (*n* = 38; Table 4), agreement regarding the presence of active small bowel inflammation was modest, with Cohen's kappa of 0.11 (95% CI −0.19 to 0.41; *p* = 0.03) and an overall concordance rate of 52.6%. Using VCE as the reference, MRE showed a sensitivity of 69.2%, specificity of 44%, positive predictive value (PPV) of 39.1%, and negative predictive value (NPV) of 73.3% for active disease. Agreement between the combined MRE/IUS assessment and VCE (*n* = 46) was even lower, with a kappa of −0.07 (95% CI −0.19 to 0.34; *p* = 0.012) and a concordance rate of 50% (Table 4). Not all children had imaging or complete biomarker data within the predefined time window around VCE examination, and data are presented for those with available data as indicated. Figure 3 illustrates the discordance in the presence or absence of inflammation across VCE, imaging, and biochemical definitions, underscoring that many patients classified as being in remission by FC or imaging still had active mucosal inflammation on VCE. ​Within tests performed in children with CD (Table 3, *n* = 42), VCE detected small bowel inflammation in 35 examinations (83%). MRE was abnormal in 10 of 27 studies (37%), and IUS was abnormal in 8 of 18 (44%), both lower than the proportion with active inflammation on VCE. Agreement between VCE and cross-sectional imaging for active disease in this cohort remained limited.

**Table 4:** Diagnostic agreement between cross-sectional imaging (magnetic resonance enterography and intestinal ultrasound) and video capsule endoscopy in children evaluated for suspected or established Crohn’s disease.

Comparison	N	Agreement rate (%)	Normal–Normal (TN)	Abnormal–Abnormal (TP)	Normal→Abnormal (FP)	Abnormal→ Normal (FN)	NPV	PPV	Specificity	Sensitivity	Cohen's *κ* (95% CI)	McNemar (*P* value)
MRE vs. VCE	38	52.6	11	9	14	4	73.3	39.1	44	69.2	0.11 (−0.19 to 0.41)	0.034
MRE + IUS vs. VCE	46	50	12	11	18	5	70.6	37.9	40	68.8	0.07 (−0.19 to 0.34)	0.012

MRE, magnetic resonance enterography; IUS, intestinal ultrasound; VCE, video capsule endoscopy; TN, true negative; TP, true positive; FP, false positive; FN, false negative; NPV, negative predictive value; PPV, positive predictive value; CI, confidence interval.

## Discussion

In this single-center pediatric cohort, video capsule endoscopy proved to be a safe, high-yield modality for small bowel evaluation, particularly in children investigated for CD. VCE frequently yielded clinically relevant findings that were not detected by prior endoscopy or cross-sectional imaging. A new diagnosis of Crohn's disease was established in 13 of 28 children (46%), and previously unrecognized L4 upper gastrointestinal involvement was identified in 10 of 23 children with known disease (43%). Overall, VCE findings directly influenced clinical management in 23 of 36 patients (64%), most commonly prompting treatment initiation or escalation, and in one case guiding surgical resection for a severe stricture. Our findings align with previous pediatric and adult series demonstrating that capsule endoscopy has a superior sensitivity for proximal small-bowel pathology and specifically active inflammation compared with MRE and ultrasound, especially in isolated small-bowel disease (11, 12, 14, 15). In our cohort, VCE mainly detects additional mucosal lesions not identified by cross-sectional imaging and provides complementary information, and these results should be interpreted cautiously, given the retrospective design. The marked increase in L4 classification after VCE underscores how standard ileocolonoscopy and imaging may underestimate upper GI involvement, which in turn can influence risk stratification and treatment decisions. limited, with low *κ* values and significant McNemar asymmetry. Using VCE as the reference, cross-sectional imaging showed modest sensitivity and limited specificity, indicating that reliance on imaging alone may miss active mucosal disease in some children and that these modalities do not target the same disease compartment, as they are optimized for transmural and extraluminal rather than purely mucosal inflammation. Therefore, the modest agreement between VCE and MRE/IUS likely arises, at least in part, from these different biological and anatomical targets, supporting the interpretation that VCE and cross-sectional imaging provide complementary rather than interchangeable information. Similarly, CRP and fecal calprotectin may classify patients as being in remission despite ongoing inflammatory activity on VCE, reinforcing that biomarkers cannot reliably substitute direct mucosal visualization. Capsule retention was rare and occurred in a child with previously unrecognized stricturing disease. In this case, the retained capsule localized the stricture, allowed careful pre-operative planning, and the patient underwent elective resection two months later without adverse events or need for urgent intervention.

Beyond its diagnostic performance, VCE also offers important advantages in patient experience. Adult surveys have shown that most individuals prefer VCE over colonoscopy or MRE (1), mainly due to greater comfort and lower procedure-related anxiety, suggesting that wider use of VCE in children may similarly improve acceptance of small-bowel evaluation and longitudinal monitoring. In our cohort, however, 25% of procedures required endoscopic, sedated capsule placement, which may attenuate some of these advantages.

This study has several limitations. Its retrospective, single-center design may limit generalizability and introduce selection bias, and not all children had complete biomarker or imaging data around the time of VCE, reducing the power of some concordance analyses. The study is also limited by the use of VCE as the primary comparator for small-bowel mucosal disease rather than an independent gold standard. Because VCE was performed only when clinically indicated, often after other tests had already raised concern for small-bowel disease, the high diagnostic and management yield may overestimate the impact that would be seen if capsule endoscopy were applied more uniformly.

Taken together, our results support positioning VCE as a complementary tool to ileocolonoscopy, biomarkers, and cross-sectional imaging in pediatric CD, particularly when there is diagnostic uncertainty, discordant findings, or a need to confirm deep small bowel remission. Integrating VCE into treat-to-target strategies, alongside biomarkers and cross-sectional imaging, may help refine mucosal healing goals in pediatric CD, but this approach requires validation in prospective studies.

In conclusion, VCE is a safe, well-tolerated modality for evaluating pediatric small bowel disease, particularly in Crohn's disease. Its limited concordance with other imaging modalities underscores its complementary role and supports the broader integration of VCE into routine pediatric small-bowel assessment.

## Data Availability

The raw data supporting the conclusions of this article will be made available by the authors, without undue reservation.
